# Identification and Survey of a Novel Avian Coronavirus in Ducks

**DOI:** 10.1371/journal.pone.0072918

**Published:** 2013-08-30

**Authors:** Gui-Qian Chen, Qing-Ye Zhuang, Kai-Cheng Wang, Shuo Liu, Jian-Zhong Shao, Wen-Ming Jiang, Guang-Yu Hou, Jin-Ping Li, Jian-Min Yu, Yi-Ping Li, Ji-Ming Chen

**Affiliations:** 1 Institute of Cell Biology and Genetics, College of Life Sciences, Zhejiang University, Hangzhou, China; 2 The Laboratory of Avian Disease Surveillance, China Animal Health and Epidemiology Center, Qingdao, China; The University of Hong Kong, China

## Abstract

The rapid discovery of novel viruses using next generation sequencing (NGS) technologies including DNA-Seq and RNA-Seq, has greatly expanded our understanding of viral diversity in recent years. The timely identification of novel viruses using NGS technologies is also important for us to control emerging infectious diseases caused by novel viruses. In this study, we identified a novel duck coronavirus (CoV), distinct with chicken infectious bronchitis virus (IBV), using RNA-Seq. The novel duck-specific CoV was a potential novel species within the genus *Gammacoronavirus*, as indicated by sequences of three regions in the viral 1b gene. We also performed a survey of CoVs in domestic fowls in China using reverse-transcription polymerase chain reaction (RT-PCR), targeting the viral nucleocapsid (N) gene. A total of 102 CoV positives were identified through the survey. Phylogenetic analysis of the viral N sequences suggested that CoVs in domestic fowls have diverged into several region-specific or host-specific clades or subclades in the world, and IBVs can infect ducks, geese and pigeons, although they mainly circulate in chickens. Moreover, this study provided novel data supporting the notion that some host-specific CoVs other than IBVs circulate in ducks, geese and pigeons, and indicated that the novel duck-specific CoV identified through RNA-Seq in this study is genetically closer to some CoVs circulating in wild water fowls. Taken together, this study shed new insight into the diversity, distribution, evolution and control of avian CoVs.

## Introduction

RNA viruses are of great diversity, and they are the etiological agents of many important human and animal infectious diseases, including influenza, rabies, several types of infectious hepatitis, severe acute respiratory syndrome (SARS), classical swine fever, rinderpest and avian infectious bronchitis (IB) [1]–[10]. Timely identification of RNA viruses is of great significance in the diagnosis, treatment, control and prevention of human and animal infectious diseases [11]. However, this work is quite difficult if the RNA virus has not been discovered in the past. For example, it took multiple years to identify hepatitis C virus as the etiological agent of most non-A non-B infectious hepatitis in the 1980s [12]. It also took several months for scientists to identify the SARS coronavirus (CoV) in 2003 [4], [5].

The development of the next generation sequencing (NGS) technologies in recent years has helped us make great progress in rapid identification of novel RNA viruses via RNA-Seq [13]. RNA-Seq is able to simultaneously sequence millions of DNA fragments reversely transcribed from RNA using random primers. Usually, most RNA-Seq reads are from cellular RNA, but some may be from RNA virus genomes, if the sequenced samples have been properly processed to minimize the quantity of cellular RNA before reverse transcription, and thus RNA-Seq could be used to identify RNA viruses [14]–[17].

In this study, we obtained some sequences of a novel CoV through a pilot detection of RNA viruses in duck feces using RNA-Seq. The phylogenetic relationship and distribution of this duck CoV was subsequently studied.

Currently, the subfamily *Coronavirinae* in the family *Coronaviridae* covers four genera, *Alpha-*, *Beta-*, *Gamma-* and *Deltacoronavirus* [1]. The first three correspond to the former nonofficial “groups” 1, 2 and 3. SARS CoV is located within the genus *Betacoronavirus* [18], [19]. All known CoVs detected from domestic fowls are members of the genus *Gammacoronavirus*, while some CoVs detected from some wild birds are members of the genus *Deltacoronavirus* [20], [21].

Currently, the genus *Gammacoronavirus* is represented by the avian coronaviruses which include infectious bronchitis virus (IBV) as well as similar viruses isolated from pheasants and peafowl, turkey coronavirus, and IBV-like viruses from teal, geese, pigeons and ducks. Several coronaviruses from mammals have also been placed in the *Gammacoronavirus* genus, including a Beluga whale coronavirus SW1 [21], [22], [24]. IBV circulates widely in many regions in the world, and can cause acute and highly contagious respiratory diseases in chickens of all ages and diminish egg production in hens [23]. Control of the disease is mainly based on vaccination, but antigenic changes of the virus may result in incomplete protection through vaccination. Although chickens are the primary natural hosts of IBVs, several studies have identified IBV-like viruses in turkeys, ducks, geese and pigeons [24]–[27]. Due to the fact that few genomic sequences of these IBV-like viruses circulating in domestic fowls other than chickens or turkeys have been identified, the taxonomy of these IBV-like viruses remains uncertain. For example, complete or partial genomic sequences of only one duck IBV-like virus have been reported in GenBank. Additionally, partial genomic sequences of 39 goose IBV-like viruses isolated in only one country (Norway) have been reported in GenBank, and 33 of the sequences are only 208 bp in length covering a short and conserved region in the viral 1b gene [28].

As mentioned above, we have found some clues which indicated a potential novel avian CoV in ducks. We further performed a molecular survey of CoVs in domestic fowls in China, to determine the prevalence of avian CoVs in apparently healthy domestic fowls and the distribution of the novel duck CoV in domestic fowls.

**Figure 1:**
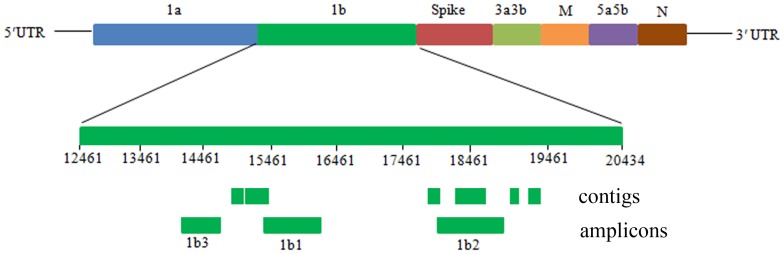
The genome structure of avian CoV and positions of the contigs and amplicons detected in this study.

Regarding molecular surveys of pathogenic viruses, it is important to select a proper region in the viral genomes as the detection target. If the target region in the viral genome is too conserved, the sequences of the region may be of little use in phylogenetic analysis, due to limited mutations in the conserved sequence. On the other side, if the region in the viral genome is highly variable, the detection may be insensitive in identifying the target virus, although the sequences of the region may be useful in the phylogenetic analysis. Considering these factors, we selected a moderately conserved region in the genomes of IBV and IBV-like viruses, namely a region of the viral nucleocapsid (N) gene, rather than the highly variable spike (S) gene or the highly conserved replicase genes, 1a or 1b (Figure 1), as the target of the survey in this study.

## Results

### Metagenomic Analysis of RNA-Seq Results

A total of 13,781,274 sequence reads were obtained through the RNA-Seq detection of the pooled duck feces sample. Of them, 22 sequence reads were homologous with IBV genes through the standalone BLAST search in the downloaded nucleotide database, indicating the existence of a CoV in the duck feces sample. The duck CoV was designated as DK/CH/ZJ2012 and abbreviated as DK2012-CoV below. These 22 sequences reads formed six contigs corresponding to the viral 1b gene, as showed by Figure 1. Totally, the six contigs were 1,626 bp in length, and harbored 15.70% different nucleotide residues compared to the corresponding sequences of the unique duck CoV, DK/CH/HN/ZZ2004 (abbreviated as DK2004-CoV below), in GenBank (Access No. JF699752).

### Amplification of Three Regions in the Viral 1b Gene

Five pairs of primers were designed based on the sequences of the six contigs and conserved regions in avian CoVs and synthesized for gene amplification. Two pairs of these primers and another pair of conserved primers reported previously [29], (Table 1) were successful in the amplification and sequencing of three regions of the viral 1b genes. The three regions were designated as 1b1 (848 bp), 1b2 (736 bp), and 1b3 (560 bp), corresponding to positions 15058–15921, 18164–18927, 14235–14756, respectively, in the genome (Figure 1).

**Table 1:** The RT-PCR primers successfully used in amplification of the viral 1b gene.

Primer pair	Sense	Sequence
IBV-1b1	Upper	5′-GGATATAGACAAGGGTAGTAA-3′
	Down	5′-ACAAAACACTCTGGTACCAT-3′
IBV-1b2	Upper	5'-GGTGGTAGTCTGTATGTGAA-3'
	Down	5'-GGATACCGCTTATAACACT-3′
IBV-1b3	Upper	5′-TGGGWTGGGAYTAYCCWAARTGYGA-3′
	Down	5′-GCATWGTRTGYTGNGARCARAATTC-3′

**Figure 2:**
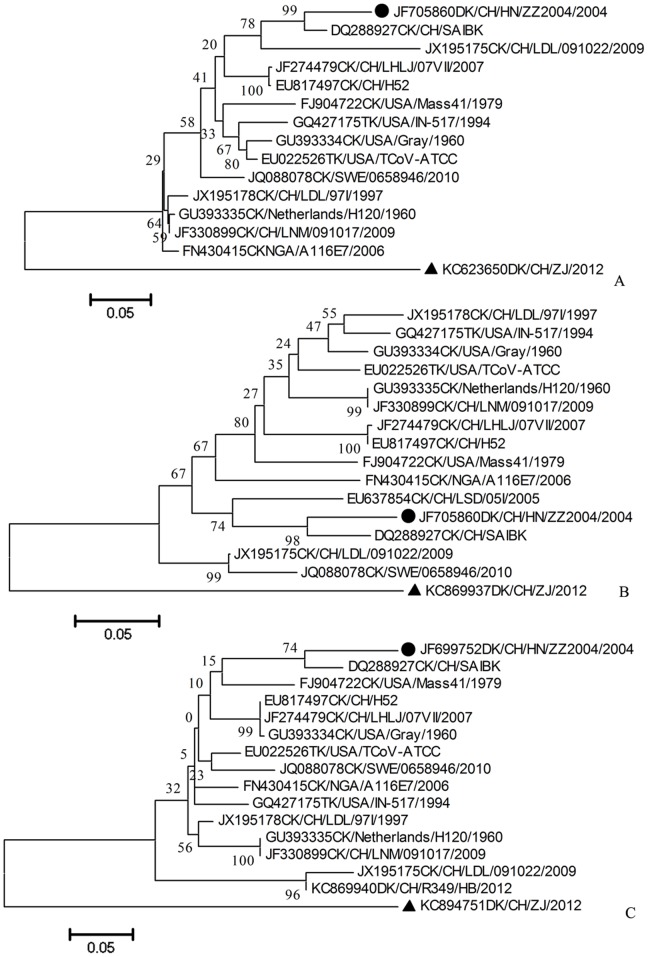
Phylogenetic relationships of two duck CoVs and 13 IBVs based on the 1b1 (A), 1b2 (B) and 1b3 (C) regions in the viral 1b gene. The GenBank accession numbers were given before the corresponding strain designations where CK, DK, CH were the abbreviations of chicken, duck and China. Two CoVs detected from ducks were marked in triangles and circles. Bootstrap values were given at the relevant nodes.

**Figure 3:**
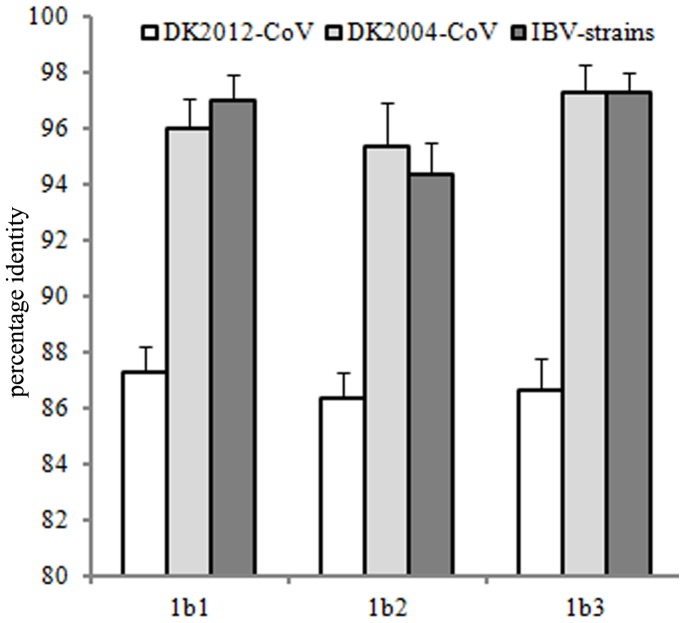
The percentage identities between two duck CoVs and 13 IBVs based on their amino acid sequences in the 1b1, 1b2 and 1b3 regions in the viral 1b gene.

The phylogenetic relationships of the sequenced viral 1b gene of DK2012-CoV, along with DK2004-CoV and 13 randomly selected IBVs, were calculated using the maximum likelihood method under the substitution model of T92+G, which was of the lowest Bayesian Information Criterion (BIC) scores and considered to describe the substitution pattern the best [30]. The results suggested that the 1b1, 1b2 and 1b3 sequences were distinct with their counterparts of IBVs (Figure 2). The mean identities in the amino acid sequences between DK2012-CoV and the 13 IBVs were 87.30% in 1b1, 86.37% in 1b2 and 86.66% in 1b3. By contrast, the mean amino acid sequence identities between DK2004-CoV and the 13 IBVs were 95.99% in 1b1, 95.35% in 1b2, and 97.31% in 1b3, similar to the mean amino acid sequence identities within the 13 IBVs, 97% in 1b1, 94.38% in 1b2, and 97.31% in 1b3 (Figure 3). These data suggested that DK2004-CoV and IBVs belong to the same species, and DK2012-CoV identified in this study represents a potential novel species, according to the nomenclature standard of CoVs that CoVs of amino acid sequence identity <90% in the domains of the viral replicase genes (1a and 1b) could be assigned to a novel species [1].

### Survey of Avian CoVs Targeting the Viral N Gene Sequences

A total of 2614 samples from domestic fowls (2002 from chickens, 502 from ducks, 55 from geese, and 55 from other birds) were collected and detected in the survey. Among them, 90 positive samples (85 from chickens, 4 from ducks, 1 from goose) were identified through the RT-PCR targeting the viral N gene. In general, the prevalence of the virus was approximately 4.25% in chickens, 0.80% in ducks and 1.82% in geese. The survey samples were collected from 150 flocks in 16 provinces, and positive samples were identified in 19.33% of the 150 flocks and 75.00% of the 16 provinces. In addition, among the 34 clinical specimens collected from chicken farms, 11 positive samples were identified.

The viral N gene sequences of a total of 102 CoVs (90 from the positive survey samples, 11 from the positive clinical specimens, one from the RNA-Seq sample) were analyzed with their counterparts (>400 bp) of avian CoVs (*n = *428) belonging to the Genus *Gammacoronavirus* in GenBank on February 20, 2013. Their phylogenetic relationships were firstly calculated using the simple neighbor-joining method, and then some sequences with identity >95% from the same host species, the same country and the same period, such as those from chickens in the USA during the period 1965–1978, were randomly deleted for the subsequent analysis in order to minimize the calculation and demonstration difficulty. Phylogenetic relationships of the remaining 122 representative sequences were calculated using the maximum likelihood method under the substitution model of T92+G, which was of the lowest BIC scores [30].

**Figure 4:**
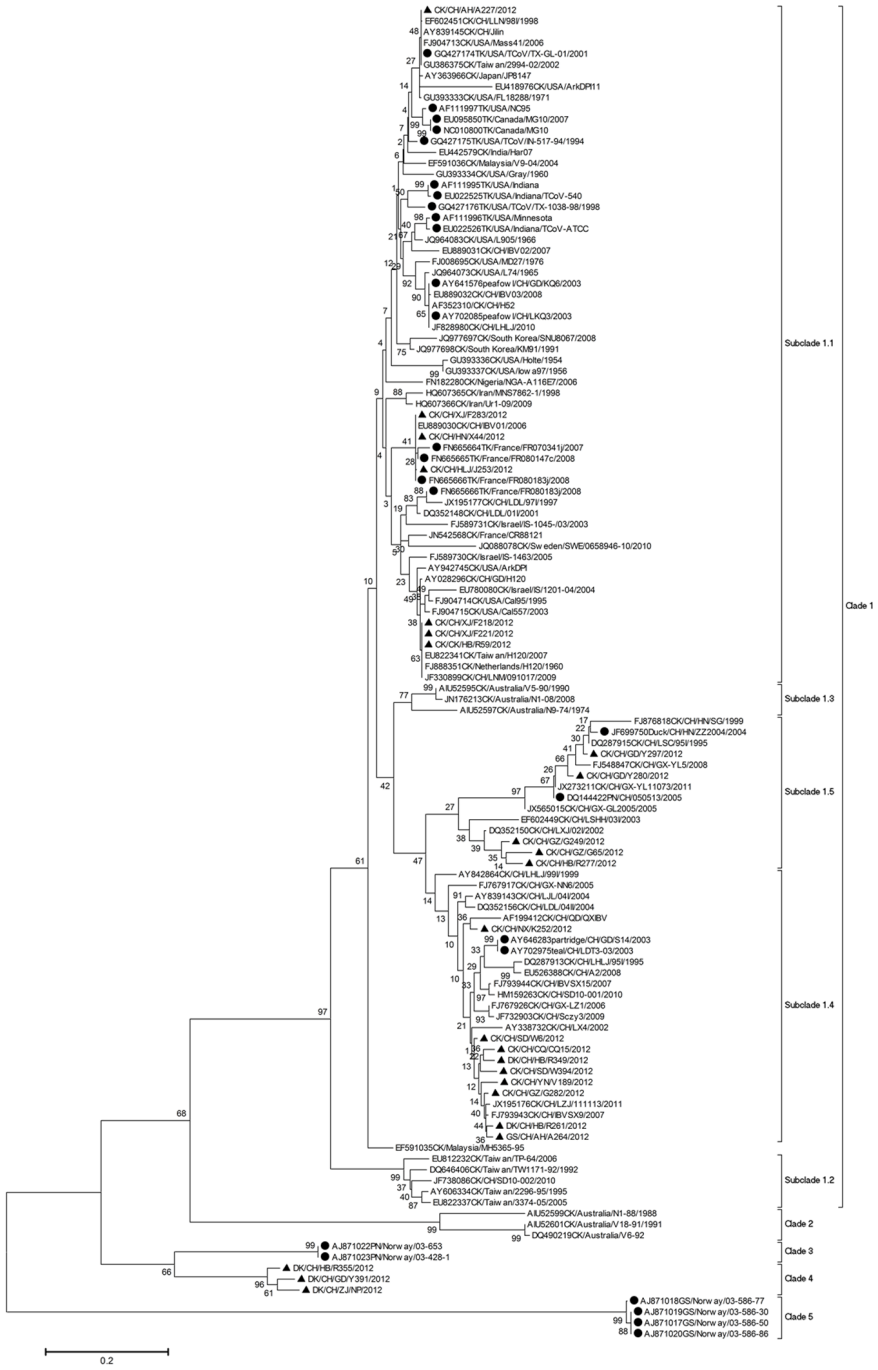
Phylogenetic relationships of representative avian CoVs based on their N gene sequences. The GenBank accession numbers were given before the corresponding strain designations where CK, DK, TK, GS, PN, CH were the abbreviations of chicken, duck, turkey, goose, pigeon and China. The CoVs reported by this study were marked with triangles, and the CoVs from birds other than chickens were marked with circles except for those reported by this study. Bootstrap values were given at the relevant nodes.

The phylogenetic tree (Figure 4) suggested that the all the avian CoVs can be classified into five clades. Clade 1 covered those common IBVs distributed in many countries including the USA, China, Iran, Israel, India, Malaysia, Australia, Japan, South Korea, Sweden, France, Nigeria, etc. from the 1960s to the 2010s. Clade 2 covered some IBVs detected in Australia from the 1980s to the 1990s. Clade 3 covered the novel duck CoVs reported in this study. Clade 4 covered some pigeon CoVs detected in Norway in 2003. Clade 5 covered some goose CoVs detected in Norway in 2003. Clade 1 could be further divided into Subclades 1.1–1.5, corresponding to IBVs detected in many countries including China from the 1950s to the 2010s (subclade 1.1), some IBVs detected in Taiwan from the 1990s to the 2010s (subclade 1.2), some IBVs detected in Australia from the 1970s to the 2000s (subclade 1.3), many IBVs detected in China (subclade 1.4), and some IBVs detected in China (subclade 1.5), respectively.

The mean of genetic distance among the clades is 0.4897 (SD = 0.0381). The mean of genetic distance among the subclades is 0.1773 (SD = 0.0147). The mean of genetic distance within the clades is 0.0929 (SD = 0.0095). The mean of genetic distance within the subclades is 0.0732 (SD = 0.0073).

We further calculated the phylogenetic distribution of the 530 avian CoVs whose N gene sequences have been analyzed in this study using the neighbor-joining method. Of these 530 CoVs, 375 were from China and the remaining 155 were from other countries. 74.84% of the 155 avian CoVs from other countries in Europe, Asia, North America and the Pacific, belonged to Subclade 1.1, indicating it was the predominant subclade in the world. Subclade 1.1 also covered 23.47% of Chinese strains. In contrast, among the 375 Chinese strains, most (53.87%) belonged to Subclade 1.4, 23.47% belonged to Subclade 1.1, 14.40% belonged to Subclade 1.6, and the remaining 8.27% belonged to other Clades or subclades. Therefore, subclade 1.4 was assumed to be predominant in China.

Figure 4 exhibited that avian CoVs have likely formed some clusters specific to some host species or some regions. For example, Clades 3, 4 and 5 were likely specific to pigeons, ducks and geese. Clade 2 and Subclade 1.2 were largely specific to Australia and Taiwan. Subclades 1.4 and 1.5 were specific to mainland China.

Figure 4 demonstrated that all the CoVs from turkeys and some CoVs from pigeons, ducks and geese, including two from ducks and one from goose identified in this survey, were similar to many chicken IBVs within the same Clade 1. The chicken IBVs identified through this survey from China all belonged to Clade 1 and were distributed in Subclades 1.3, 1.4 and 1.5. A total of four duck CoVs were identified through the survey. Two of them were genetically closer to chicken IBVs in Clade 1, and the other two were genetically closer to the duck-specific Clade 4 along with the virus DK2012-CoV which was identified through RNA-Seq. The two duck CoVs sharing the same Clade with DK2012-CoV were supposed to be within the same potential novel species with DK2012-CoV.

### Phylogenetic Analysis of the Viral 1b3 Sequences

An interesting study on the prevalence and phylogeny of CoVs in wild birds has been reported also targeting the 1b3 region [31]. Consequently, sequences of the 1b3 region, rather than of the 1b1 and 1b2 regions, of multiple species of wild birds were available in GenBank. We analyzed their phylogenetic relationships with DK2012-CoV reported by this study using the maximum likelihood method under the substitution model of T92+G, which was of the lowest BIC scores [30].

**Figure 5:**
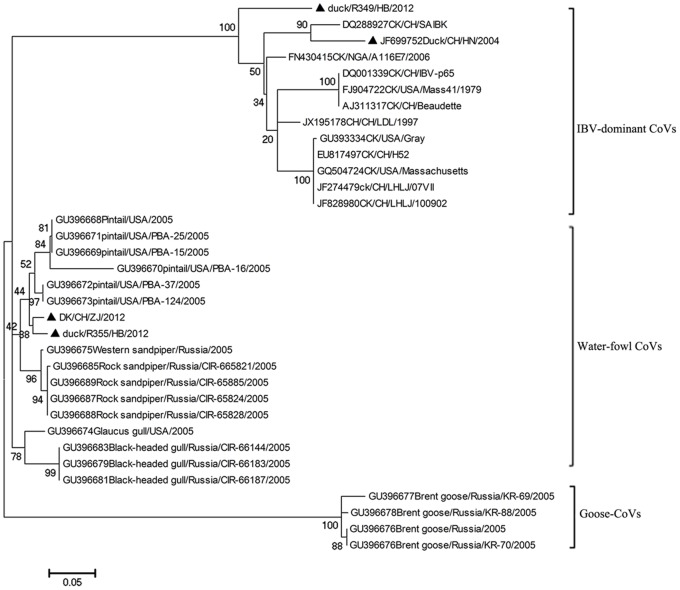
Phylogenetic relationships of some CoVs based on their 1b3 sequences. Duck CoVs were marked with triangles. Bootstrap values were given at the relevant nodes.

As Figure 5 indicated, the DK2012-CoV belonged to the same clade along with some CoVs identified from water fowls of pintails, sandpipers and gulls, suggesting that they may share the same phylogeny which was different from the ones corresponding to IBVs and some goose CoVs. However, it remains unknown whether the CoVs from pintails, sandpipers and gulls belong to the same potential novel species along with DK2012-CoV, due to that sequences of only one region in the genomes were available for these wild water fowl viruses. The previous report has indicated that the phylogeny covering DK2012-CoV were prevalent in wild water fowls in the Bering Strait Area [31].

As consistent with Figure 5 based on the viral N gene sequences and previous reports [32], [33], Figure 5 based on the 1b3 region also suggested that CoVs from birds have formed some host-specific clusters.

## Discussion

RNA-Seq is a powerful tool in detecting novel RNA viruses nowadays, and facilitates the studies on the evolution and surveillance of RNA viruses [34]. However, RNA-Seq remains technique-complicated. Firstly, it usually requires a considerable amount of starting RNA for cDNA library construction [15]. Secondly, during the extraction of viral RNA, it is tough to completely delete rRNA from the host and some bacteria, even though rRNA deletion products have been available to minimize the inference of rRNA of the host and some bacteria [35]–[37]. Consequently, many RNA-Seq reads belong to rRNA of cellular organisms. Thirdly, if the viral reads are highly divergent from any known viruses, no hits may be found for these viral reads. The RNA-Seq used in this study is based on Ion Torrent PGM, which is a small and relatively cheap NGS sequencer with much less throughput, compared to some other NGS sequencers, such as Hi-Seq of Illumina or Roche 454 FLX Sequencers [38]. Possibly for these factors, we have obtained only several contigs rather than the full genome of DK2012-CoV through RNA-Seq.

The genomes of CoVs are approximately 27–31 kb in length, the largest for known animal RNA viruses [39]. Rapid mutations plus large RNA genomes of CoVs create difficulties in developing effective vaccines and conducting viral genome sequencing. Therefore, even though avian CoVs frequently cause severe infections in poultry in many countries [23], studies on avian CoVs have remained limited, especially on those circulating in birds other than chickens. To our knowledge, this report presented the first large-scale epidemiological survey of avian CoVs in poultry in a large country which provided the virus prevalence in multiple species of domestic fowls. It also presented the first global phylogenetic analysis based on the viral N gene which provided a panorama view as to the diversity and distribution of avian CoVs in the world and in China. Moreover, this study has identified a potential novel species of CoVs in the genus of *Gammacoronavirus* specific to ducks or some water fowls.

The classification of these clades or subclades is largely consistent with some previous reports based on the viral S or 1b gene sequences. For example, the clades or subclades identified to be specific to some chicken IBVs in Australia (Clade 2), to some chicken IBVs in Taiwan (Subclade 1.2), to some pigeon CoVs in Norway (Clade 3), or to some goose CoVs in Norway (Clade 5), also belonged to separate phylogenies, indicated by previous reports [28], [31], [33], .

The clades or subclades of avian CoVs identified in this report are somehow host-specific or region-specific. For example, Clade 1 was largely specific to chickens, and Clade 3 was specific to pigeons, Clade 4 was specific to ducks and Clade 5 specific to geese, while Clade 2 and Subclade 1.3 specific to Australia, Subclade 1.2 largely specific to Taiwan, and Subclades 1.5 and 1.6 specific to mainland China. The host-specific clades or subclades may result from that different virus strains are of different efficiencies in replication in different hosts. This may lead to separate replication and circulation of different clades or subclades of the CoVs in different hosts, similar to reproduction isolation of higher organisms. Although the viral spike gene is directly involved in the viral receptor-binding, the replication isolation can also act on the viral N gene through genetic hitchhiking [30]. The region-specific clades or subclades may result from geographical isolation caused by the limited transmission ability of the virus and the spatial isolation of the regions (e.g. Australia and Taiwan are both separate islands). The explanation of the specificity of the clades and subclades, in turn, supports the theory that replication or reproduction isolation and geographical isolation is important in speciation [43], [44].

This study suggested that subclade 1.1 was worldwide predominant and subclade 1.4 was predominant in China. Their predominance may result from that they were of greater transmission ability than others. Vaccines targeting these predominant subclades may be better than those targeting other subclades in the relevant regions.

This study provided novel data to support that IBV is prevalent in chickens and seldom in other domestic fowls, yet IBVs can infect ducks, geese and pigeons, and that IBVs have diverged into several region-specific clades in the world. Moreover, this study also provided novel data to support that some host-specific CoVs other than IBVs circulate in ducks, geese and pigeons, and indicated that the novel duck-specific CoV identified in this study is genetically closer to some CoVs circulating in wild water fowls.

Taken together, this study shed new insight into the diversity, distribution and evolution of avian CoVs. It also provided important information for the relevant vaccine research.

## Materials and Methods

### Ethics Statement

This study was conducted according to the animal welfare guidelines of the World Organization for Animal Health [45], and approved by the Animal Welfare Committee of China Animal Health and Epidemiology Center. The feces samples from clinical dead ducks, and the swab samples from poultry farms, backyard flocks, slaughtering houses and live bird markets, and the clinical specimens from 34 chicken farms, were all collected with permission given by multiple relevant parties, including the Ministry of Agricultural of China, China Animal Health and Epidemiology Center, the relevant veterinary section in the provincial and county government, and the owners of the relevant veterinary clinics for the dead duck samples or the owners of the relevant bird flocks for other samples. The feces samples from clinical dead ducks were collected from the intestines of the ducks that died from diseases. Swab samples were collected by gently taking smears from the trachea and cloacae of the domestic fowls and then placed in a transport medium. Clinical specimens of lungs from chicken farms were collected from chickens that died from diseases.

### Sample Collection and Treatment for RNA-Seq

Feces from 52 clinical dead ducks were collected in Zhejiang province. The samples were pooled together and diluted using a nine-fold volume of phosphate buffered saline (pH 7.2). Then, the solution was centrifuged at 12,000 g at 4°C for 1 h, and the supernatant was filtered using a PVDF membrane (pore size: 0.22 um; Millipore) to further remove eukaryotic and bacterial particles. The filtered solution was then mixed at 4°C for 2 h with a 1/10 volume of 50% (w/v) PEG 6000 which was dissolved in 1.5 M NaCl. Then the solution was centrifuged at 12,000 g for 1 h at 4°C. The pellet was re-suspended into 100 µL of Hank’s balanced salt solution. To remove the naked DNA and RNA in the solution, the solution was further incubated with the cocktail of DNase and RNase enzymes consisting of 14 U of Turbo DNase (Ambion), 20 U of Benzonase (Novagen) and 20 U of RNase One (Promega) at 37°C for 3 h in 1×DNase buffer (Ambion). This was followed by the extraction of viral RNA using the QiaAmp Viral RNA Kit (Qiagen), and the viral RNA was eluted in 60 µL of the elution buffer.

### RNA-Seq Library Construction and Sequencing

The viral RNA was applied for cDNA library preparation according to the protocol reported previously, except without the step of RNA fragmentation [15]. Briefly, the RNA was reversely transcribed into first-stranded cDNA via the first adaptor. Then, the first-stranded cDNA was purified using the Ampure XP magnetic beads (Beckman Coulter). This was followed by the synthesis of the second-strand using the second adaptor. Primers from both adaptors were used to obtain the cDNA library. Then, the concentration of cDNA library and dilution factors were determined for the subsequent emulsion PCR. The emulsion PCR was performed using the Ion One Touch machine and Ion RNA-Seq Kit v2 (Life Technologies). Positive ion sphere particles of the emulsion PCR products were enriched through multiple steps by the Ion ES machine, and then the positive spheres were transferred to Ion Torrent PGM for sequencing.

### Analysis of RNA-Seq Data

Sequences in the NCBI nonredundant nucleotide database (NT) similar to the sequence reads of the RNA-Seq were searched using the standalone BLASTn [46]. The BLASTn results were parsed with the software MetaGenome Analyzer (MEGAN) [47]. Sequence reads classified into viruses through the MEGAN software were extracted and assembled using the CLC genomics workbench. The resultant contigs were further analyzed using the online BLAST [46], and they were used for design of RT-PCR primers in order to obtain more genomic sequences of viruses identified through the RNA-Seq.

### Survey Sample Collection and Treatment

In November, 2012, 2614 swab samples were collected randomly from domestic fowls in poultry farms, backyard flocks, slaughtering houses and live bird markets in 17 provinces in China. The swab samples were collected by taking smears from the trachea and cloacae of the fowls and placed in a transport medium. The samples were clarified by centrifugation at 10,000 g for 5 min, and the supernatants were inoculated in 10-day-old specific-pathogen-free (SPF) chicken embryonated eggs via the allantoic sac route. The SPF embryonated eggs were purchased from Shandong Healthtec Laboratory Animal Breeding Company (Jinan, China). The inoculated eggs were further incubated for 2 d, and checked twice each day during the incubation period. The dead ones were picked out and stored in a refrigerator. After the incubation period, the allantoic fluids of the live embryos and the dead eggs were investigated further by RT-PCR, as described below. In addition, clinical specimens were collected from 34 chicken farms in Zhejiang and Shandong provinces and treated using the same methods described above.

### RT-PCR Detection and Sequencing for the Survey

RNA was extracted using an RNeasy Mini Kit (Qiagen), and amplified with the QIAGEN One Step RT-PCR Kit (Qiagen), using the primers 5'-TGAATCGTGGTAGGAGTG-3' (upper) and 5'-TTCCTCTTGTAGCAGGTCT-3' (down). The primers flank a 573-nucleotide region in the viral N gene, and the primers are conserved in all known avian CoVs within the genus *Gammacoronavirus*. RT-PCR was performed in a 50-µL reaction system with incubation at 42°C for 30 min and denaturation at 94°C for 2 min, followed by 30 cycles at 94°C for 30 s, 50°C for 30 s and 72°C for 1 min. RT-PCR products were purified with an agarose gel DNA extraction kit (Sangon, Shanghai, China), and directly sequenced using a Perkin-Elmer model 377 XL DNA sequencer with the RT-PCR primers from both directions.

### Phylogenetic Analysis and Genetic Distance Calculation

Phylogenetic relationships of the viral N gene sequences obtained through this study were analyzed using the software package MEGA 5.03 [48], along with their counterparts of all avian CoVs of known isolation countries and known isolation years within the genus *Gammacoronavirus* in GenBank. These sequences were aligned using the program Clustal X covered by MEGA 5.03. Then, the best substitution model of the aligned sequences was determined as the one with the lowest Bayesian-Information-Criterion scores calculated by MEGA 5.03. This was followed by phylogenetic analysis according the best substitution model [30]. Bootstrap values were calculated out of 1000 replicates. All the phylogenetic trees were calculated by two separate groups to avoid manual errors. Phylogenetic relationships of the viral 1b gene sequences in this study was performed in the same way except that only several reference sequences selected randomly from GenBank were involved in the analysis. Genetic distances were calculated using the same parameters used for the phylogenetic analysis.

### GenBank Accession Numbers

All the sequences reported herein have been submitted to the GenBank database with the accession numbers: KC623570–KC623649 and KC786227–KC786249 for the viral N gene sequences, KC623650, KC869937, KC869938–KC869940 and KC894751 for the 1b1, 1b2 and 1b3 sequences, and KC869933–KC869936 for the sequences of the RNA-Seq contigs (>200 bp).

### Strain Designation and Nucleotide Numbering

Sequences of avian CoVs reported previously or by this study were designated or re-designated in the order of the GenBank accession number, hosts, collection country, collection places (only for most Chinese strains), collection years. Genomic positions were numbered according to the DK2004-CoV with the GenBank database accession number JF705860.
